# Diagnostic Challenges of OHVIRA Syndrome—A Case Report

**DOI:** 10.3390/jcm15010190

**Published:** 2025-12-26

**Authors:** Paulina Tomecka, Adam Jagodziński, Justyna Łuczak, Łukasz Waszczuk, Marek Murawski

**Affiliations:** 1 Faculty of Medicine, Wroclaw Medical University, 50-367 Wroclaw, Poland; 2 Clinical Department of Gynecologic Surgery and Oncology, Wroclaw Medical University, Borowska 213, 50-556 Wroclaw, Poland; marek.murawski@umw.edu.pl; 3 Pediatric Surgery and Urology Department, Wroclaw Medical University, Borowska 213, 50-556 Wroclaw, Poland; justyna.luczak@umw.edu.pl; 4 Department of General and Interventional Radiology and Neuroradiology, Wroclaw Medical University, Borowska 213, 50-556 Wroclaw, Poland; lukasz.waszczuk@umw.edu.pl

**Keywords:** OHVIRA syndrome, uterine duplication, unilateral vaginal obstruction, renal agenesis, congenital anomalies of the female reproductive system

## Abstract

**Background and Clinical Significance**: Herlyn–Werner–Wunderlich (HWW) syndrome, also known as OHVIRA syndrome (Obstructed HemiVagina and Ipsilateral Renal Anomaly), is a rare congenital anomaly of the female urogenital system characterized by uterine duplication, unilateral vaginal obstruction, and renal agenesis on the same side. The condition often remains undiagnosed until adolescence, when it presents with palpable pelvic mass, dysmenorrhea, and chronic pelvic pain. **Case report**: We present the case of a 13-year-old female patient diagnosed with OHVIRA syndrome following imaging studies. Surgical treatment involved incision of the vaginal septum and evacuation of accumulated blood, leading to symptom resolution and restoration of reproductive tract patency. **Conclusions**: This article discusses the clinical characteristics, diagnostic challenges, and the importance of early surgical intervention, emphasizing the necessity of considering this syndrome in the differential diagnosis of adolescent females with cyclic abdominal pain and renal anomalies. Early diagnosis and treatment can prevent severe health complications and improve both patients’ quality of life and fertility.

## 1. Introduction

Nearly 7% of girls are born with congenital abnormalities affecting female reproductive system [1]. These anomalies may appear alone or along with the defects in the kidneys, bladder, or anorectal region [2]. Herlyn–Werner–Wunderlich (HWW) syndrome is a rare urogenital disorder linked to Müllerian and mesonephric duct malformations, defined by a classic triad of developmental defects: uterine duplication, unilateral vaginal obstruction, and ipsilateral renal agenesis [3]. Although HWW syndrome has traditionally been considered extremely rare, with some clinical reports indicating an incidence of approximately 1 in 1,000,000 females, it is increasingly recognized as underdiagnosed [4]. The syndrome is estimated to represent 0.1–3.8% of Müllerian duct anomalies, though its true prevalence within the general population remains undetermined [5,6]. This underrecognition is likely due to variable clinical presentation and the delayed onset of symptoms, which often become apparent only after menarche [7]. Notably, a retrospective case series identified HWW syndrome as the most commonly diagnosed obstructive Müllerian anomaly in adolescents presenting after menarche [8].

HWW syndrome, also called Obstructed HemiVagina and Ipsilateral Renal Anomaly (OHVIRA) syndrome, has been increasingly recognized in medical literature [9,10]. This terminology is now more commonly used due to reports of cases involving uterine anomalies beyond the typical uterine duplication, including normal, septate, and other structural variations. The exact pathogenesis of OHVIRA syndrome remains unclear. However, genetic, environmental, and hormonal factors are thought to play a role in its development [11]. Based on recent classification, OHVIRA is categorized into two subtypes: type 1, characterized by complete vaginal obstruction, and type 2, involving partial obstruction [12].

HWW syndrome often remains undiagnosed in childhood and typically occurs after menarche with symptoms of reproductive dysfunction [13]. The average age at diagnosis is 14 years [14]. Common clinical features include chronic pelvic pain, dysmenorrhea, and a palpable pelvic mass, often resulting from hematocolpos or hematometra due to vaginal or uterine obstruction [15]. This article presents a case of OHVIRA syndrome in a 13-year-old patient, emphasizing diagnostic difficulties and therapeutic considerations.

## 2. Case Presentation

A 13-year-old girl was referred to the Department of Pediatric Surgery and Urology, University Hospital in Wrocław, for urgent evaluation due to a two-month history of dull abdominal pain. The patient reported no fever, nausea, or vomiting but complained of nocturnal hot flashes.

An abdominal ultrasound performed at the regional hospital failed to visualize the right kidney. The findings were suspicious for a vaginal septum, with a differential diagnosis including an ovarian cyst or endometriosis. Physical examination revealed a soft abdomen with no palpable pathological masses or peritoneal signs, and her BMI was within the normal range (19.4). However, deep palpation elicited tenderness in the lower abdomen and both iliac fossae. Chełmoński and Goldflam signs were negative, effectively excluding gallbladder and renal pathology, with the Chełmoński sign assessed by tapping the right costal margin and the Goldflam sign by percussion of the costovertebral angle.

**Figure 1:**
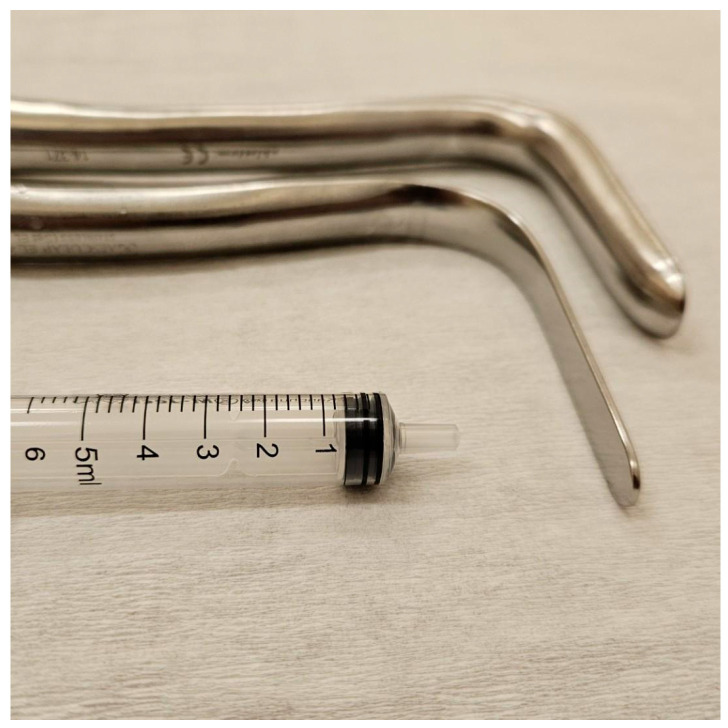
Metal vaginal speculum used in the examination, designed for young or virgin patients and those with a narrow vagina. It features a straight, narrow design with smooth edges, allowing gentle and controlled insertion with minimal risk of trauma. The speculum enables visualization of the vaginal walls and cervix during gynecologic examinations or biopsies. Recommended procedural steps include warming the speculum, adequate lubrication, angled insertion toward the cervix, and slow opening to avoid injury.

The patient had no history of chronic medical conditions, and her only previous surgery was tonsillectomy at age 10. A gynecologic examination was subsequently performed. She had menarche at age 11 and had since experienced regular but painful menstrual cycles. Internal examination revealed a normally developed vulva with a vaginal entrance that allowed digital examination and the insertion of a virgin speculum. The virgin vaginal speculum used during the examination is shown in Figure 1. Upon speculum insertion, a bulging, tense area was observed on the right side of the vaginal wall, raising suspicion of vaginal duplication with right-sided hematocolpos. The vaginal portion of the cervix was not visible in the unobstructed part of the vagina.

**Figure 2:**
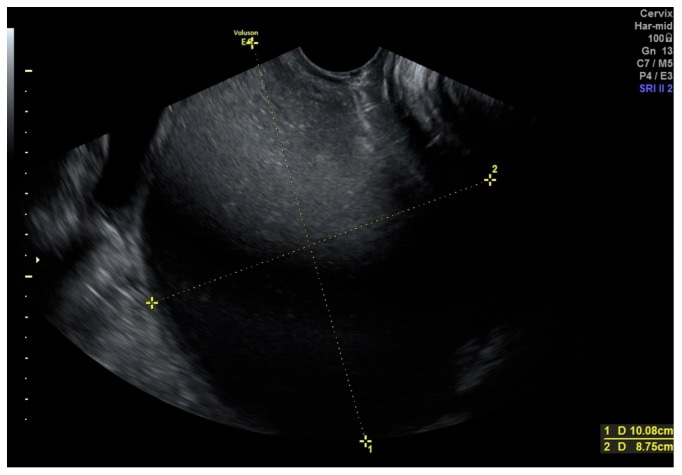
Transvaginal view demonstrating a large fluid-containing structure (>10 cm) accessed via the vaginal vestibule.

**Figure 3:**
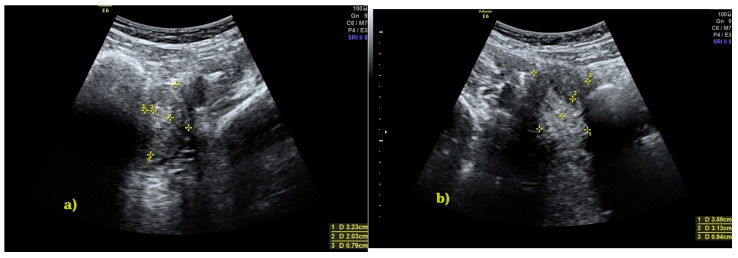
Transabdominal ultrasound showing two uterine bodies: (**a**) left uterine body, 32 × 20 mm; (**b**) right uterine body, 36 × 31 mm.

In transvaginal ultrasound, performed from the vestibular area, a cystic mass over 10 cm in diameter was visualized, with echogenicity suggestive of hemolyzed blood (Figure 2). A transabdominal ultrasound confirmed the presence of this structure, while both ovaries exhibited a normal follicular pattern. The left ovary measured 34 × 27 mm, and the right ovary diameter was 38 × 26 mm. Two separate uterine bodies were visualized: the left uterus measured 32 × 20 mm with an endometrial thickness of approximately 8 mm, while the right uterus measured 36 × 31 mm with an endometrial thickness of 9 mm (Figure 3). The left kidney was identified in its normal position, whereas the right kidney was absent. These findings suggested a diagnosis of OHVIRA syndrome.

**Figure 4:**
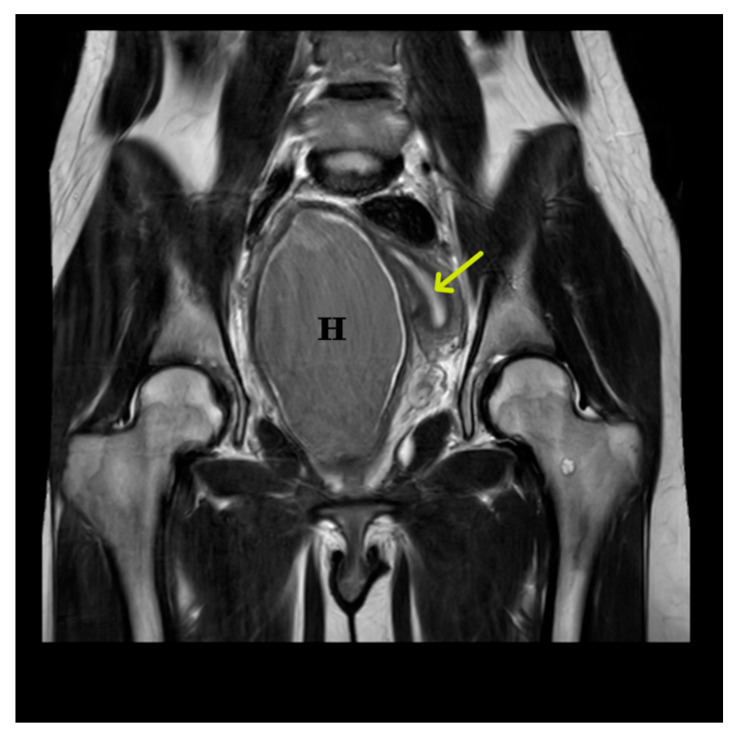
Preoperative coronal T2-weighted MRI of the pelvis, acquired prior to surgical incision of the hematocolpos. The examination was performed using a standard pelvic MRI protocol. The image demonstrated a markedly dilated right hemivagina containing material with intermediate-to-low T2 signal, consistent with hematocolpos (H), measuring approximately 68 × 77 × 120 mm (estimated volume ~330 mL). Both vaginal canals converged at the level of the vestibule. The vaginal septum was presumed to be longitudinal; however, its evaluation was limited by the presence of hematocolpos (H). Uterus didelphys was present, with two distinct uterine bodies: the right uterine body was superficially located in the right pelvic fossa, whereas the left uterine body was situated deeper and more posteriorly within the pelvis (yellow arrow). The urinary bladder and the visualized portion of the colon showed no apparent pathological changes.

**Figure 5:**
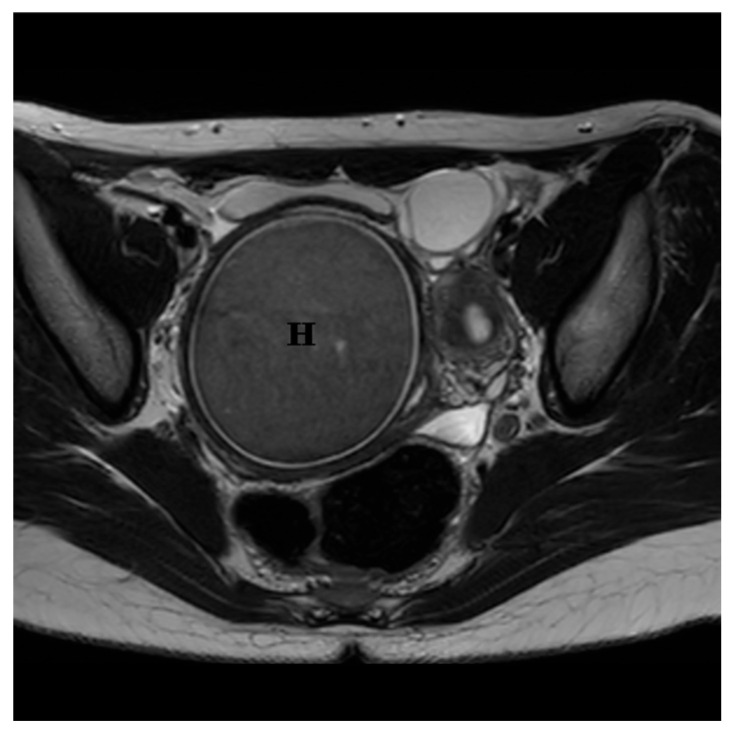
Axial T2-weighted MRI, obtained prior to hematocolpos incision, demonstrated a well-circumscribed, homogeneously hypointense collection within the right hemivagina, consistent with hematocolpos (H). The image also demonstrated an associated Müllerian duct anomaly classified as U3C2V2 (uterus didelphys with duplicated cervices and vaginas). Both ovaries were visualized and appeared unremarkable, without focal lesions. No lymphadenopathy or increased free pelvic fluid was observed.

Further imaging with pelvic MRI confirmed a congenital anomaly of the reproductive tract, characterized by a double uterus, cervical divergence, and vaginal duplication. Two separate uterine bodies were present: the right uterus was superficially located in the right pelvic fossa with an endometrial thickness of up to 10 mm, while the left uterus lay deeper and more posteriorly. The left cervix was elongated (up to 48 mm) but exhibited no focal abnormalities. The two vaginal canals converged at the level of the vestibule. A hemorrhagic collection was identified in the right vaginal canal, measuring approximately 68 × 77 × 120 mm, with an estimated volume of 330 mL. Assessment of the vaginal septum was limited due to the presence of hematocolpos. The parametria are unremarkable and rotated to the left. MRI images of the genital tract are shown in Figure 4 and Figure 5.

Based on comprehensive clinical and radiological findings, a final diagnosis of OHVIRA syndrome was established. The patient was scheduled for a right-sided vaginal septum incision. The procedure was performed using diathermy, with a longitudinal 3 cm incision made in the vaginal septum to restore patency of the right vaginal canal. Approximately 300 mL of viscous, hemolyzed blood was evacuated. The procedure was successful with no complications. On the first postoperative day, speculum examination confirmed the patency of the vaginal incision and ruled out active bleeding. A bimanual pelvic examination revealed both uterine bodies to be palpable, painless, and mobile. The vagina was well-formed, and the entrance to the right vaginal canal remained patent, with a diameter of approximately 1.5 cm. At one month, the patency of the incision was confirmed.

## 3. Discussion

OHVIRA syndrome is typically characterized by a double uterus, a unilaterally obstructed vagina, and ipsilateral renal agenesis. Additionally, variations such as a bicornuate uterus, septate uterus, polycystic renal dysplasia, or ureteral ectopia have also been reported [16]. The diagnosis of OHVIRA syndrome is primarily based on evaluating the causes of dysmenorrhea and pelvic pain after menarche. In adolescent patients, other potential causes of abdominal pain, including ovarian lesions, acute appendicitis, and pelvic inflammatory disease, should be excluded. In reported cases, an ovarian cyst or endometriosis is often initially suspected. Although anatomical variations exist in OHVIRA syndrome, pain is typically unilateral and persists for several days following menstruation [17]. Most cases are diagnosed after menarche due to complications related to uterine and vaginal obstruction, urogenital infections, and pelvic adhesions. The severity of symptoms depends on the degree of vaginal obstruction. In patients with partial obstruction, symptoms such as purulent discharge or signs of vaginal infection may not develop until several years after menarche [18]. One retrospective study found that patients remain asymptomatic until puberty, highlighting the difficulty of early detection [19]. Many patients have to undergo surgery due to misdiagnosis or inadequate treatment of complications [20,21]. Studies have shown that up to 22% of patients with OHVIRA syndrome undergo unnecessary surgery due to an incorrect diagnosis of ovarian cysts [8]. Accurate diagnosis is difficult because patients usually have regular menstrual cycles. When cyclical menstrual pain occurs, analgesics, nonsteroidal anti-inflammatory drugs (NSAIDs), or oral contraceptives are often prescribed, which may delay diagnosis and prevent consideration of OHVIRA syndrome [22].

Pelvic ultrasound is the diagnostic tool of choice because of its cost-effectiveness, ease of use, and lack of ionizing radiation. However, accuracy depends largely on the skill and experience of the examiner [23]. Ultrasound can detect hematocolpos, hematometra, duplicated uterus, and unilateral renal agenesis but cannot directly visualize the vaginal septum [24]. MRI is considered the gold standard because it allows for detailed three-dimensional visualization of the female reproductive tract and other pelvic structures [25]. Over the first 60 years after the syndrome was initially described, only 115 cases were documented. In comparison, the past two decades alone have yielded a further 170 cases, largely reported from tertiary-care centers. This increase may be due to the widespread use of ultrasound and MRI, which have enabled the timely and accurate diagnosis of Müllerian anomalies [14]. Diagnostic accuracy can be improved by performing a renal examination in patients with abdominal and pelvic pain, as more than 30% of women with unilateral renal agenesis also have Müllerian anomalies [26]. Studies have shown that approximately 92% of patients diagnosed with OHVIRA syndrome have renal agenesis, while the remaining 8% have polycystic kidney disease [27].

Surgery is the mainstay of treatment for OHVIRA syndrome. Vaginal septum excision is usually performed intravaginally; however, laparoscopic surgery may be more effective if the septum is proximally located [28]. Routine laparoscopy is not always necessary, but patients should be monitored for complications, such as endometriosis, which occurs in approximately 14% of cases [29]. Postoperative follow-up is crucial to detect and manage complications such as dyspareunia, vaginal stenosis, and recurrent vaginal obstruction.

Although long-term follow-up data are not yet available for our 13-year-old patient, available evidence indicates that early surgical intervention in patients with OHVIRA is associated with favorable reproductive outcomes. Previous studies report that thirteen out of 21 women attempting to conceive after correction of an obstructed hemivagina achieved pregnancy [30]. Overall, patients with HWWS demonstrate positive reproductive and obstetric outcomes, with approximately 87% achieving pregnancy and around 62% experiencing uncomplicated deliveries [31]. Systematic reviews suggest that uterine malformations such as a bicornuate uterus do not appear to reduce fertility compared with normal uteri, although they may be associated with an increased risk of miscarriage or preterm birth [32]. Obstetric complications reported in patients with HWWS include preeclampsia, preterm delivery, fetal breech presentation, and a high cesarean section rate, underscoring the need for careful prenatal monitoring [30]. In addition, some adolescents with obstructive reproductive tract anomalies may develop endometriosis, which could adversely affect future fertility. Kapczuk et al. reported endometriosis in six of 50 patients who had no evidence of the disease at the time of corrective surgery but later developed persistent dysmenorrhea resistant to NSAIDs, highlighting the importance of long-term gynecological surveillance [33].

Taken together, these data suggest that our patient has a reasonable chance of future fertility following early surgical management; however, the potential risks of endometriosis and obstetric complications emphasize the necessity of regular long-term gynecologic and obstetric follow-up.

## 4. Conclusions

OHVIRA syndrome should be considered in the differential diagnosis of patients with cyclic abdominal pain and those with renal agenesis or other urinary tract abnormalities. Early diagnosis of this rare disease is essential to ensure appropriate care and prevent complications. For patients with OHVIRA syndrome, it is important to decide on surgical treatment as early as possible. This helps prevent complications due to persistent vaginal obstruction, such as pyometra or tubal abscesses. Surgical incision of the vaginal septum and drainage of blood accumulated in the vagina not only minimizes the risk of complications, but also increases the patient’s chances of preserving fertility in the future. Therefore, early surgical intervention to confirm the diagnosis of OHVIRA syndrome is essential to improve the patient’s quality of life and minimize the risk of serious health and obstetric complications.

## Data Availability

The original contributions presented in this study are included in the article. Further inquiries can be directed to the corresponding authors.
